# Effect of Inadequate Sleep on Frequent Mental Distress

**DOI:** 10.5888/pcd18.200573

**Published:** 2021-06-17

**Authors:** Amanda Blackwelder, Mikhail Hoskins, Larissa Huber

**Affiliations:** 1 University of North Carolina at Charlotte, Charlotte, North Carolina

## Abstract

**Introduction:**

One-third of US adults report sleeping less than the recommended amount, and approximately 20% live with a mental illness. The objective of our study was to examine the association between inadequate sleep and frequent mental distress in a population-based sample of US adults.

**Methods:**

We conducted a cross-sectional study by using 2018 Behavioral Risk Factor Surveillance System (BRFSS) data that included 273,695 US adults aged 18 to 64. Inadequate sleep was defined as 6 hours or less in a given night, and frequent mental distress was defined as self-reporting 14 days of mental health status as “not good” within the last month. We used weighted logistic regression to calculate odds ratios (ORs) and 95% CIs.

**Results:**

Thirteen percent of study participants experienced inadequate sleep, and 14.1% experienced frequent mental distress. Participants who averaged 6 hours or less of sleep per night were about 2.5 times more likely to have frequent mental distress when controlling for confounders (OR, 2.52; 95% CI, 2.32–2.73) than those who slept more than 6 hours.

**Conclusion:**

Inadequate sleep was associated with significantly increased odds of frequent mental distress. Our findings suggest that further research is necessary to evaluate the temporal relationship between inadequate sleep and frequent mental distress.

**Summary**

**What is already known on this topic?**

One-third of US adults report that they sleep less than the recommended amount, and approximately 20% have received a diagnosis of a mental illness. The link between inadequate sleep and mental distress has been viewed historically as a symptom–disease association with sleep inadequacies deriving from preexisting mental distress.

**What is added by this report?**

We examined the association between inadequate sleep and frequent mental distress in a diverse, population-based sample of adults aged 18 to 65.

**What are the implications for public health practice?**

By identifying the correlation between inadequate sleep and frequent mental distress we can better understand this relationship as a risk factor instead of a symptom–disease relationship.

## Introduction

Poor mental health is common in the US. Nearly 1 in 5 US adults live with mental illness (1). Furthermore, an estimated 50% of all Americans will be diagnosed with a mental illness or disorder at some point in their life (1,2). Mental health illness includes many different conditions and symptoms, such as anxiety, depression, stress, and other psychological illnesses. Moderate and severe mental disorders that need psychological treatment require regular visits to a health care provider, thus lowering workplace productivity (3). Furthermore, depression, schizophrenia, and bipolar disorder are risk factors for coronary heart disease, hypertension, diabetes, dyslipidemia, metabolic syndrome, obesity, stroke, and substance abuse disorders (3,4). Depression and anxiety alone cost over $1 trillion annually for medications, outpatient and primary care visits, and inpatient care (3,4).

The Centers for Disease Control and Prevention (CDC) and the American Academy of Sleep Medicine emphasize the importance of an adequate night’s sleep, which is defined as 7 or more hours per night with no upper limit (5,6). Anything less than this amount may lead to the development of various chronic diseases. More than one-third of the US population does not get adequate sleep (5). The people that most often get inadequate sleep are Native Hawaiian/Pacific Islander people, non-Hispanic Black people, and multiracial people (6). Those who most often get adequate sleep are married people and people with a college degree or more.

Studies have demonstrated an association between inadequate sleep and frequent mental distress (7,8), and sleep deprivation causes substantial negative health outcomes (4). The link between inadequate sleep and frequent mental distress has been viewed historically as a symptom–disease association with sleep inadequacies deriving from preexisting mental distress (9). However, at least 1 study researched the opposite hypothesis, evaluating frequent mental distress leading to a lack of sleep (10). These studies found that in certain populations, risk for inadequate sleep is increased if a person is experiencing depression or anxiety. Most current research on the potential association between inadequate sleep and mental distress focuses on a specifically defined group, such as college students, nurses, or people with diagnosed sleep disorders (9,11,12). Furthermore, current research focuses primarily on diagnosed mental health disorders (4,8). The purpose of our study was to examine the association between inadequate sleep and frequent mental distress in a diverse, population-based sample of adults aged 18 to 64.

## Methods

We used 2018 Behavioral Risk Factor Surveillance System (BRFSS) data to analyze the association between sleep and self-reported mental distress. BRFSS is a cross-sectional survey that uses a standardized questionnaire to collect prevalence data regarding risk behaviors and preventive behavioral health practices among adult US residents (13). Participants self-report information during telephone interviews conducted by trained personnel. Interviewers make calls for interviews 7 days a week during the day and evening (14). BRFSS raw data, which are collected during the survey, are submitted to CDC each year for processing and are made available to researchers the following calendar year through annual reports available on the CDC website (https://www.cdc.gov/brfss/index.html). BRFSS is conducted in all 50 states, the District of Columbia, and 3 US territories. Noninstitutionalized adults aged 18 or older are eligible to complete the BRFSS survey (15). Over 400,000 adults are interviewed each year. The land line response rate for BRFSS is 53.3%, and the cellular telephone response rate is 43.3% (16). A total of 437,436 people completed the BRFSS survey in 2018. After excluding those participants who were not aged 18 to 64 (n = 160,115) and those who did not have information on frequent mental distress (n = 3,626), 273,695 survey participants remained for analysis.

The survey question used to identify the exposure variable of interest reads, “On average, how many hours of sleep do you get in a 24-hour period?” Participants were asked to provide a value from 1 to 24 hours. Sleep values were recorded as whole numbers, and values greater than 30 minutes were rounded up per BRFSS coding. Inadequate sleep was defined as 6 hours or less of sleep in a given 24-hour period, which is 1 hour less than the minimum recommended number of hours of sleep for adults (5,17,18). We chose this definition of inadequate sleep because the rounding done by BRFSS personnel could have created situations where people who actually had inadequate sleep were classified as having the minimum recommended hours. Furthermore, previous studies also defined inadequate sleep as 6 hours or less per night (5,17). Thus, using this same definition allows for better comparison across studies.

The survey question selected to identify the outcome of interest, frequent mental distress, was “Now thinking about your mental health, which includes stress, depression, and problems with emotions, for how many days during the past 30 days was your mental health not good?” (13). The responses were recorded as the number of days (ie, 1–30 days). Frequent mental distress was considered present if 14 or more days were reported as mental health “not good” in the previous month. This definition was based on recommendations from previous studies on frequent mental distress (19) and guidance from the American Psychiatric Association on the necessary duration of symptoms to diagnose depression (20).

Potential confounders were selected on the basis of prior research and included age, race/ethnicity, sex, current smoking status, binge drinking, marital status, education, income, and loss of insurance (21–28). Study participants were asked the following question regarding alcohol consumption: “One drink is equivalent to a 12-ounce beer, a 5-ounce glass of wine, or a drink with one shot of liquor. During the past 30 days, on the days when you drank, about how many drinks did you drink on the average?” (13). We used this information to create a dichotomous binge drinking variable; in the past 30 days on the days when they drank, men who had 5 or more drinks and women who had 4 or more drinks were classified as binge drinkers (29). To assess current smoking, participants were asked, “Do you now smoke cigarettes every day, some days, or not at all?” Ultimately, this variable was dichotomized into people who smoked every day or some days and people who did not smoke at all.

A primary univariate analysis was performed to obtain frequencies and weighted percentages of the exposure, outcome, and potential confounders at the *P* < .20 level. We used logistic regression to assess the association between self-reported sleep and frequent mental distress and to identify other risk factors for frequent mental distress. Multivariate logistic regression was used to obtain the odds ratio for the association between inadequate sleep and frequent mental distress while adjusting for potential confounders. A backward elimination approach was used to retain confounders at the *P* < .05 level. Ultimately, age, marital status, income, smoking status, and education level were identified as confounders. Because of the complex sampling design used by BRFSS, weighted analyses were performed using Stata version 15.1 (StataCorp LLC).

## Results

Most study participants were non-Hispanic White (59.1%), female (50.2%), married (49.3%), and had at least a high school diploma (87.4%) (Table 1). Most participants reported that they had adequate nightly sleep (87.0%), and 14.1% experienced frequent mental distress (≥14 d/mo). Mean hours of sleep per 24-hour period were similar across age groups (18–34: 6.9 h; 35–49: 6.8 h; 50–64: 6.9 h).

**Table 1:** Demographic Characteristics of Participants (N = 273,695) Experiencing Frequent Mental Distress, Behavioral Risk Factor Surveillance System, 2018

Variable	Unweighted n^a^	Weighted %
**Average^b^ sleep**		
Adequate	239,750	87.0
Inadequate	33,945	13.0
**Days mental health not good**		
Normal, <14 d/mo	235,570	85.9
High, ≥14 d/mo	38,125	14.1
**Sex**		
Female	143,771	50.2
Male	129,480	49.8
**Age, y**		
18–34	71,614	38.3
35–49	79,602	29.5
50–64	122,479	32.2
**Race/ethnicity**		
Non-Hispanic White	193,757	59.1
Non-Hispanic Black	25,117	12.5
Asian	8,138	5.8
American Indian/Alaska Native	5,843	1.1
Hispanic	30,725	19.3
Other races	10,115	2.2
**Education**		
College degree or above	104,228	28.6
High school diploma, GED, or some college	149,023	58.8
No high school diploma	19,911	12.6
**Marital status**		
Married	142,638	49.3
Divorced/separated	56,972	18.7
Widowed/never married	72,748	32.1
**Annual household income, $**		
<35,000	65,453	30.8
35,000–75,000	66,469	28.7
>75,000	91,722	40.5
**Binge drank^c^ in past 30 days**		
No	96,053	64.5
Yes	47,478	35.5
**Smoke**		
No	58,416	53.6
Yes	47,318	46.4
**Lost health coverage within past year**		
No	28,314	91.1
Yes	2,270	8.9

a Some totals may not equal the total number of participants because of missing data.

b Inadequate average sleep was defined as 6 hours or less in a 24-hour period.

c In the past 30 days on the days when they drank, men who had 5 or more drinks and women who had 4 or more drinks.

People with inadequate sleep had nearly a threefold increased odds of frequent mental distress compared with those who had adequate sleep, and this finding was significant (OR, 2.67; 95% CI, 2.51–2.84) (Table 2). Participants who were divorced/separated/widowed had twice the odds of frequent mental distress compared with study participants who were married (OR, 2.14; 95% CI, 2.01–2.29). There was a dose–response association between education level and frequent mental distress. As education levels decreased, the odds of frequent mental distress increased (high school diploma, GED, associate degree, or no university degree: OR, 2.06; 95% CI, 1.95–2.18; no high school diploma: OR, 3.35; 95% CI, 3.06–3.67).

**Table 2:** Association Between Selected Variables and Frequent Mental Distress Among Participants (N = 273,695), Behavioral Risk Factor Surveillance System, 2018

Variable	Weighted Odds Ratio (95% CI)
**Average^a^ sleep**	
Adequate	1 [Reference]
Inadequate	2.67 (2.51–2.84)^b^
**Sex**	
Female	1 [Reference]
Male	0.91 (0.87–0.96)^b^
**Age, y**	
18–34	1 [Reference]
35–49	1.00 (0.95–1.06)
50–64	1.27 (1.20–1.34)^b^
**Race/ethnicity**	
Non-Hispanic White	1 [Reference]
Non-Hispanic Black	1.12 (1.04–1.21)^b^
Asian	0.52 (0.44–0.62)^b^
American Indian/Alaska Native	1.61 (1.35–1.90)^b^
Hispanic	1.05 (0.96–1.13)
Other races	1.35 (1.21–1.51)^b^
**Education**	
College degree or above	1 [Reference]
High school diploma, GED, or some college	2.06 (1.95–2.18)^b^
No high school diploma	3.35 (3.06–3.67)^b^
**Marital status**	
Married	1 [Reference]
Divorced/separated/	2.14 (2.01–2.29)^b^
Widowed/never married	1.29 (1.22–1.36)^b^
**Annual household income, $**	
<35,000	2.71 (2.54–2.91)^b^
35,000–75,000	1.56 (1.45–1.68)^b^
>75,000	1 [Reference]
**Binge drank^c^ in past 30 days**	
No	1 [Reference]
Yes	1.21 (1.13–1.30)^b^
**Smoke**	
No	1 [Reference]
Yes	1.80 (1.68–1.93)^b^
**Lost health coverage within past year**	
No	1 [Reference]
Yes	1.40 (1.17–1.69)^b^

a Inadequate average sleep was defined as 6 hours or less in a 24-hour period.

b Significant at *P* < .20.

c In the past 30 days on the days when they drank, men who had 5 or more drinks and women who had 4 or more drinks.

After adjustment for age, marital status, income, smoking status, and education level, the inadequate sleep–frequent mental distress association was attenuated but remained significant. Participants with inadequate sleep had nearly 2.5 times increased odds of frequent mental distress compared with those with adequate sleep (OR, 2.52; 95% CI, 2.32–2.73; *P* < .001).

## Discussion

In our population-based study of US adults, inadequate sleep was associated with significantly increased odds of mental distress after controlling for confounding variables. Our findings align with previous research with the caveat that prior research has often looked at sleep as the outcome (8). Because our study used a large sample of adults and excluded only those who did not respond to qualifying questions, our results further confirm a potential association between inadequate sleep and mental health in a broader population.

Our study findings suggest an association between inadequate sleep and frequent mental distress. Because BRFSS is a cross-sectional study design, determining the true temporal sequence is not possible. Previous research has not closely examined the association between inadequate sleep as a risk factor for frequent mental distress. However, inadequate sleep has been linked to poor biological measures, including hypertension, anemia, and dyslipidemia (7). Low amounts of sleep and the attributed chronic conditions could possibly have a negative impact on depressive symptoms (7).

Limitations to this study include the potential for nondifferential misclassification of both the exposure and outcome variables; failure to recall information or misunderstanding questions asked possibly resulted in inaccurate responses. Also, self-reporting of mental distress is subjective. People may differ in their self-reporting and interpretation of what is “not good” for mental health. Furthermore, the use of a telephone interview could possibly influence self-reporting of mental distress. However, research demonstrates that the reporting of mental health information does not differ between face-to-face interviews and telephone interviews (31,32). In some instances, telephone interviews reduced embarrassment to participants when discussing mental health. We used a cut point of 6 hours to determine inadequate sleep rather than 7 hours, which is the minimum recommended hours of sleep for adults. We reran our model using 7 hours as the cut point for inadequate sleep, and our findings were of similar magnitude and remained significant. Given our aforementioned concerns that the rounding done by BRFSS personnel as it relates to the sleep duration variable could have incorrectly classified participants, we ultimately decided to retain our 6-hour cut point. In addition, because this definition of inadequate sleep has been used by others, it allows for better comparison across studies (5,17). Regardless, any nondifferential misclassification in our study would likely bias the results toward the null. Because we used a secondary data source, we were limited to the questions asked in the BRFSS survey. Thus, confounding by variables not measured in the BRFSS was possible. Selection bias is possible given that the response rate for BRFSS was 53.3% for landline responses and 43.3% for cellular telephone responses (15). The extent to which participation in BRFSS would be related to inadequate sleep and frequent mental distress is unknown; however, BRFSS is widely considered to be a valid and reliable measure of mental health and health behaviors (33).

Our study had numerous strengths. Information bias is unlikely because of the use of trained interviewers and standardization of interview methods. The exposure question may capture sleep data with more precision because it asks how many hours the participant slept in a 24-hour period. Thus, naps are included in the reporting. In addition, the wording enables the sleeping habits of people who do not work traditional day-time jobs to be more accurately reported. Establishing hours slept in a 24-hour period is consistent with prior research, giving a continuity of comparison across studies (4,7,8). Finally, our study included all participants aged 18 to 64 and did not focus only on those with preexisting conditions or on populations at risk for inadequate sleep (4,8). Thus, given the large sample size and the complex sampling design used by BRFSS, our findings are likely generalizable to adults living in the US.

Because one-third of the US population is not attaining adequate sleep, our findings warrant further research to expand on the true association between inadequate sleep and frequent mental distress (5). Thorough clinical assessment of sleep by age and length and quality of sleep could strengthen the measurement of the exposure. More thorough follow-up questions related to mental distress, including clinical diagnoses, may allow for a clearer evaluation of the temporal sequence between inadequate sleep and mental distress.
